# A Robust Machine Learning Framework Built Upon Molecular Representations Predicts CYP450 Inhibition: Toward Precision in Drug Repurposing

**DOI:** 10.1089/omi.2023.0075

**Published:** 2023-07-19

**Authors:** Sotiris Ouzounis, Vasilis Panagiotopoulos, Vivi Bafiti, Panagiotis Zoumpoulakis, Dionisis Cavouras, Ioannis Kalatzis, Minos-Timotheos Matsoukas, Theodora Katsila

**Affiliations:** ^1^ Institute of Chemical Biology, National Hellenic Research Foundation, Athens, Greece.; ^2^ Department of Biomedical Engineering, University of West Attica, Egaleo, Greece.; ^3^ Cloudpharm PC, Athens, Greece.; ^4^ Department of Food Science and Technology, University of West Attica, Egaleo, Greece.

**Keywords:** cytochrome P450, ADME-Tox, drug repurposing, predictive computational models, quantitative structure-activity relationships, machine learning

## Abstract

Human cytochrome P450 (CYP450) enzymes play a crucial role in drug metabolism and pharmacokinetics. CYP450 inhibition can lead to toxicity, in particular when drugs are co-administered with other drugs and xenobiotics or in the case of polypharmacy. Predicting CYP450 inhibition is also important for rational drug discovery and development, and precision in drug repurposing. In this overarching context, digital transformation of drug discovery and development, for example, using machine and deep learning approaches, offers prospects for prediction of CYP450 inhibition through computational models. We report here the development of a majority-voting machine learning framework to classify inhibitors and noninhibitors for seven major human liver CYP450 isoforms (CYP1A2, CYP2A6, CYP2B6, CYP2C9, CYP2C19, CYP2D6, and CYP3A4). For the machine learning models reported herein, we employed interaction fingerprints that were derived from molecular docking simulations, thus adding an additional layer of information for protein-ligand interactions. The proposed machine learning framework is based on the structure of the binding site of isoforms to produce predictions beyond previously reported approaches. Also, we carried out a comparative analysis so as to identify which representation of test compounds (molecular descriptors, molecular fingerprints, or protein-ligand interaction fingerprints) affects the predictive performance of the models. This work underlines the ways in which the structure of the enzyme catalytic site influences machine learning predictions and the need for robust frameworks toward better-informed predictions.

## Introduction

Xenobiotic metabolism is concerned with biotransformation through which the structure of a chemical substance is altered to facilitate excretion from the body. There are multiple phases of biotransformation, which occur either simultaneously or sequentially (Basak et al., 2022), and involve enzymatic catalysis during phase I and phase II drug metabolism. Cytochrome P450 (CYP450) enzymes play a central role in phase I of xenobiotic metabolism. CYP450 enzymes mostly catalyze oxidative reactions to biotransform lipophilic xenobiotics so as to produce water-soluble molecules for efficient metabolism (Testa et al., 2012).

The CYP450 system in humans is a group of heme-containing membrane-bound enzymes that are primarily located in either the smooth endoplasmic reticulum or the mitochondrial inner membrane of hepatocytes, while they are also found in the brain, skin, lung, kidney, and gut mucosa (Guengerich, 2020). The seven major human liver CYP450 isoforms are 1A2, 2A6, 2B6, 2C9, 2C19, 2D6, and 3A4, which also bear genomic variants. The isoforms of CYP1, CYP2, and CYP3 families account for 80% of interethnic and interindividual variations upon xenobiotic administration (Zanger and Schwab, 2013).

CYP450 isoforms consist of a 400 to 500 amino acid sequence and their active site comprises a heme cofactor. Due to their three-dimensional structure, CYP450s can adapt to heterogeneous substrates covering a diverse set of sizes and shapes affecting their selectivity. Of note, their ability to transform xenobiotics is mostly affected by their structural differences in their binding site (Tyzack and Kirchmair, 2019). This is also their key characteristic when drug-drug interactions and toxicity events are taken into account and monitored by regulatory agencies (Sudsakorn et al., 2020).

*In vitro* and *in vivo* ADME-Tox profiling remain a critical piece in drug discovery and development or drug repurposing. At the same time, such assays are expensive and time-consuming, require an expert user, cannot provide structure-activity data, and can be only applied to already synthesized chemical entities. Thus, several computational approaches that aim to predict CYP450 inhibition and improve attrition rates have been developed and applied, leaving room for improvement due to immense data variability.

Early computational approaches relied on quantitative structure-activity relationship (QSAR) models (Locuson and Wahlstrom, 2005), which lacked accuracy and generalization ability (Gleeson et al., 2007). The advent of machine learning led to QSAR methods that were enhanced by sophisticated classifiers producing robust results.

Wu et al. (2019) reported one of the most prevalent approaches that provide accurate predictions and insights for CYP450 inhibitors. The authors employed an extreme gradient boosting (XGBoost) model, among other machine and deep learning classifiers, within a diverse set of molecular descriptors and fingerprints. The ensemble model achieved an average accuracy of 90.04% on the test set, outperforming other approaches, indicating the prevalence of ensemble learning over deep learning on CYP450 inhibition data.

A more recent and novel approach was the one implemented by Qiu et al. (2022), who developed a Geometric Convolutional Neural Network (GCNN) model that utilizes a graph convolutional network with attention mechanism for feature extraction coming from ligands, while 1-D convolution is also applied for feature extraction coming from CYP450 isoforms. The extracted features in question were then concatenated and given to a fully connected layer for classification. This approach outperformed the iCYP-MFE framework (Nguyen-Vo et al., 2022), which is a similar approach that applies multitask learning. Despite such novelty in feature extraction, the GCNN model did not outperform the XGBoost model.

CYPlebrity (Plonka et al., 2021), which is provided as a module of the web service platform NERDD (Stork et al., 2020), aimed to predict CYP450 inhibitors, based on traditional machine learning algorithms for the identification of CYP450 inhibitors. CYPlebrity models are developed upon an extended applicability domain incorporating data from PubChem (Kim et al., 2023), ChEMBL (Mendez et al., 2019), and Fujitsu, ADME Database being the only web tool for CYP450 inhibition trained with the most extended dataset available today.

We report here the development of a majority-voting machine learning framework to classify inhibitors and noninhibitors for seven major human liver CYP450 isoforms (CYP1A2, CYP2A6, CYP2B6, CYP2C9, CYP2C19, CYP2D6, and CYP3A4). Predictions were based on interaction fingerprints that are produced by the interaction of CYP450 isoforms and their ligands. Molecular docking was performed to simulate binding and extract all protein-ligand interaction fingerprints. The latter were used to train our machine learning models to classify CYP450 inhibitors and noninhibitors. The proposed framework was enriched with molecular fingerprints and molecular descriptors to provide a comparative analysis among different feature types.

## Materials and Methods

### Curation and collection of datasets

Datasets for all seven major CYP450 isoforms were retrieved from PubChem. In brief, the *in vitro* data for CYP1A2, CYP2C9, CYP2C19, CYP2D6, and CYP3A4 reported in PubChem AID: 1851 were used as a training set (Veith et al., 2009) to evaluate further our framework (Supplementary Table S1). Sitagliptin does not inhibit or induce CYP1A2, CYP2A6, CYP2B6, CYP2C9, CYP2C19, CYP2D6, and CYP3A4 human CYP450 isoenzymes and hence, served as a paradigm. Next, to evaluate the robustness of our models, an external test set for each one of those five isoforms was produced based on data retrieved from assays applying luciferase-based protocols: PubChem AID: 410 (CYP1A2); PubChem AID: 883 (CYP2C9); PubChem AID: 899 (CYP2C19); PubChem AID: 891 (CYP2D6); and PubChem AID: 884 (CYP3A4).

For all test compounds (ligands) in both training and test data, key *in vitro* parameters were taken into account: potency, activity score, fit Hill slope, curve class, and fitted R^2^ (along with panel name and ID) to label test compounds as inhibitors or noninhibitors for each CYP450 isozyme in question. A test compound was annotated as “inhibitor” if its activity score was higher than 40, potency was lower or equal to 10 μM, and the curve class was equal to −1.1, −1.2, or −2.1. An annotation of “noninhibitor” was reported, if the activity score was equal to zero, its potency was higher or equal to 57 μM, and the curve class was equal to 4. Inconclusive data were removed.

For CYP2A6 and CYP2B6, there was no assay sharing a sufficient number of test compounds when PubChem was queried. Instead, CHEMBL5282 and CHEMBL4729 were included, respectively (Supplementary Tables S2, S3), while a test compound was annotated as “inhibitor” or “noninhibitor” based on the IC_50_ and Ki values provided. A test compound was annotated as “inhibitor,” if IC_50_ and Ki values were lower than 10 μM. For “noninhibitors,” IC_50_ and Ki values exceeded 20 μM. A rather limited number of experimental data were retrieved for CYP2A6 and CYP2B6 and thus, we randomly sampled 20% of the dataset in question to serve as an external test set.

### Generation of molecular descriptors and molecular fingerprints after test compound selection

Test compound (ligand) data were retrieved as SMILES from PubChem or ChEMBL. Duplicates between train and test data were eliminated. Test compounds within the range 200 to 600 Da were selected as this is the optimal range for bioactive small molecules. Multicomponent structures, usually containing salts, were processed by removing all, but the largest fragments. Next, duplicates were removed, 3D conformations were generated, and polar hydrogens were added at pH 7.4, using OpenBabel (ver. 3.1.1) (O'Boyle et al., 2011). Training and test data were handled separately and labeling was not performed until data were given to the classifiers.

For calculating molecular descriptors, the rcdk library (Guha, 2007) was employed, which is an interface for the open source java CDK (Willighagen et al., 2017) providing *n* = 320 different types of descriptors. Seven different types of molecular fingerprints were also computed using the preprocessed compounds through either the Rcpi or rcdk libraries in R: FP4 fingerprints; MACCS (Molecular ACCess System) fingerprints; Electrotopological state (E-state) fingerprints; PubChem fingerprints; Extended-Connectivity Fingerprints (ECFPs); Standard fingerprints; and Graph fingerprints.

### Molecular docking analyses of CYP450s and generation of interaction fingerprints

3D protein structures were retrieved from Protein Data Bank (PDB) (Berman et al., 2000) along with the corresponding PDB codes: 2HI4 (CYP1A2) (Sansen et al., 2007); 1Z10 (CYP2A6) (Yano et al., 2005); 4RQL (CYP2B6) (Shah et al., 2015); 5W0C (CYP2C9) (Liu et al., 2017); 4GQS (CYP2C19) (Reynald et al., 2012); 3TBG (CYP2D6) (Wang et al., 2015); and 2J0D (CYP3A4) (Ekroos and Sjögren, 2006). The enzyme structures chosen were co-crystallized with their ligand, had no mutations in their binding sites, and had low X-ray resolution, where possible. The surface area of the binding pocket of each isozyme was calculated by a two-step process.

CavityPlus (Xu et al., 2018) was used for the detection of cavities and then, the generated file was imported in PyMOL (The PyMOL Molecular Graphics System, Version 2.0 Schrödinger, LLC) to compute a series of characteristics for the surface area and cavity in question, among which the solvent accessible surface area was the most prominent. To explore further the intrinsic properties of the binding site for each isoform, structure-based pharmacophore models were designed using Pharmit (Sunseri and Koes, 2016).

For docking calculations, coordinate files were preprocessed using Auto Dock Tools (Morris et al., 2009). Water molecules were removed, same for co-crystallized ligands, excluding the heme molecule. For test compounds, 3D conformations were prepared in pdbqt file format using OpenBabel. All compounds were docked into the catalytic site of each CYP450 isoform using AutoDock Vina (Trott and Olson, 2010). Isozymes were held rigid during the docking process, while the compounds were allowed to be flexible. The grid box size was set at either 15 × 15 × 15 Å or 30 × 30 × 30 Å in the catalytic region of the heme moiety with 1.00 grid spacing, depending on the calculated cavity size for each isoform.

To compute Protein-Ligand Extended Connectivity (PLEC) fingerprints (Wójcikowski et al., 2019), the Open Drug Discovery Toolkit (oddt) python library (Wójcikowski et al., 2015) was employed. PLEC fingerprints encode protein-ligand interactions and produce a bit vector of 16,384 bits. Default parameters were used, except for “sparse” and “count_bits,” which were set to “False.” PLEC fingerprints provided a new approach for the prediction of CYP450 inhibition or no inhibition by chemical entities (test compounds). Interaction fingerprints and docking scores were used as descriptors downstream machine learning model development.

### Creation of a machine learning framework

The computed fingerprints and descriptors were processed before being fed to machine learning models. During the first phase of such processing, features with low variance and high correlation (>75%) were removed. The remaining features were subjected to feature selection by Recursive Feature Elimination (RFE) (Guyon et al., 2002). The above process was applied only in the training data, separately in each feature type and isoforms, and the resulting features were then selected from the test data to avoid data leakage. RFE-selected features were taken into account for interaction fingerprints, molecular descriptors, and molecular fingerprints.

For model training, dataset values were normalized to the range between 0 and 1. Normalization was performed on the training data per dataset and normalization parameters were then applied to transform the test data in question. The selected machine learning algorithms were based on ensemble learning, which empowers optimal discrimination between inhibitors and noninhibitors (Wu et al., 2019). Ensemble learning is the process during which a set of base learners is trained on a dataset and results from each learner are integrated through a combinatory module to provide the final prediction (Rokach, 2010). In classification problems, this combinatory module is a voting scheme.

There are two main types of ensemble learning, bagging (Breiman, 1996) and boosting (Freund and Schapire, 1997). In our framework, we employed five models (bagging, *n* = 3 and boosting, *n* = 2): the Bagged Trees method (Dietterich, 2000) and the Random Forest classifier (Liaw and Wiener, 2002) were implemented as bagging models, while the Stochastic Gradient Boosting (Friedman, 2002) model and XGBoost (Chen and Guestrin, 2016) were selected as boosting algorithms. The XGBoost classifier was used with two different types of boosters, one linear and a tree-based one. The machine learning scheme has been developed using the Caret library in R.

Our voting framework consisted of ensemble learning classifiers as base learners and used their predictions to generate a final prediction through majority voting. The voting scheme was based on majority, which means that at least three of the base learners had to agree if the test compound in question was an inhibitor or a noninhibitor. The probability of the voting scheme was computed by taking the mean value of the five probability values produced by each learner for the majority class.

### Model evaluation and data interpretation

Model hyperparameters were tuned during training and models were evaluated by k-fold cross-validation. Tuning was performed by the default option provided by the Caret R package, according to which, if z is the number of tuning parameters, a grid with size 3^z^ is automatically created. For each data type, five models were produced (each resulting in predictions for the external test data). Those predictions were used as an input for the majority voting scheme. To assess the generalization ability of all data types, the majority voting framework was also considered.

A confusion matrix was produced based on predicted and actual classes and metrics were defined by the following equations:

$$Accuracy=\frac{TP+TN}{TP+TN+FP+FN}$$

$$Sensitivity=\frac{TP}{TP+FN}$$

$$Specificity=\frac{TN}{TN+FP}$$

$$Matthewscorrelationcoefficient=\frac{TP\times TN-FP\times FN}{\sqrt{\left(TP+FP\right)\left(TP+FN\right)\left(TN+FP\right)\left(TN+FN\right)}}$$

where TP and TN refer to inhibitors and noninhibitors that are correctly identified and classified, whereas FN corresponds to inhibitors that are wrongly predicted as noninhibitors and FP denotes noninhibitors that are classified as inhibitors. We also computed the area under curve (AUC) from the receiver operating characteristic (ROC) curves produced by model predictions.

For data interpretation, the function varImp of the Caret R package was applied (models, *n* = 5 and datasets per CYP450, *n* = 23). For the Random Forest and the Stochastic Gradient Boosting algorithms, a model-dependent calculation was executed (Friedman, 2001). For the other three classifiers, a statistical approach was applied by performing an ROC curve analysis on each predictor, where a series of thresholds was applied to each variable, followed by sensitivity and specificity calculations. The AUC served as the measure of variable importance.

## Results

### The catalytic pockets of CYP1A2, CYP2A6, and CYP2B6 are smaller than those of CYP2C9, CYP2C19, CYP2D6, and CYP3A4 isoforms

**FIG. 1.**
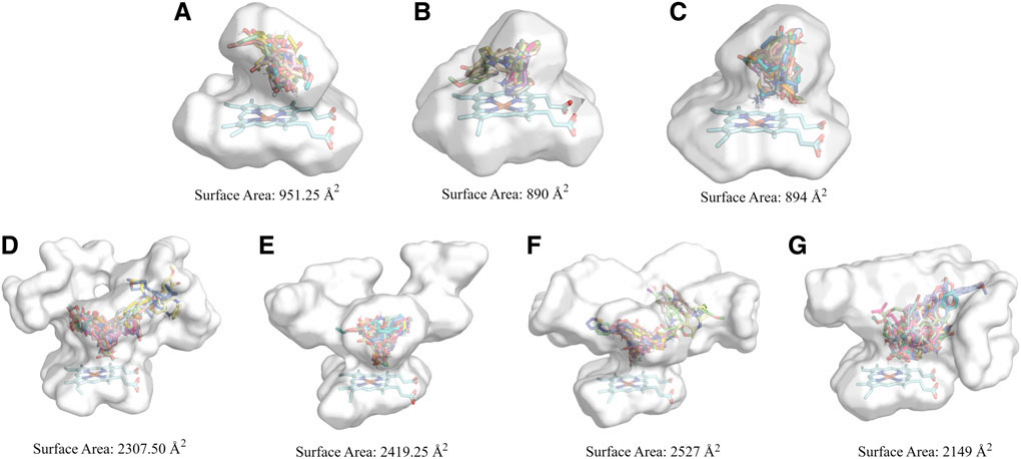
Surface areas per CYP450 isoform with a representative number of docked molecules in their binding sites. **(A)** CYP1A2, **(B)** CYP2A6, **(C)** CYP2B6, **(D)** CYP2C9, **(E)** CYP2C19, **(F)** CYP2D6, and **(G)** CYP3A4.

Before docking calculations, cavity analysis was performed, revealing that the surface area of CYP1A2, CYP2A6, and CYP2B6 had a size lower than the half surface area of CYP2C9, CYP2C19, CYP2D6, and CYP3A4 (Fig. 1). Furthermore, a structure-based pharmacophore modeling analysis was performed on the co-crystallized ligands for each of the isoforms to explore further their catalytic cavity (Supplementary Fig. S1 and Supplementary Table S4).

### Compound similarity varies and depends on the type of description

To evaluate how the calculated molecular and/or interaction fingerprints can be used best for the prediction of CYP450 inhibition, similarity analysis was performed in the training and test data of each isoforms, employing different feature types (Supplementary Fig. S2). Findings demonstrate that compound similarity varies and depends on the type of description.

Different types of compound representations provide different similarity levels for the same compounds. Molecular fingerprints provide information on the chemical nature of each test compound, while interaction fingerprints inform about protein-ligand (test compound) interactions. In the training set, interaction fingerprints for inhibitors have a higher number of compound pairs with Tanimoto similarity within the range 0.2 to 0.5, implying interactions are highly heterogeneous. For noninhibitors, similarity values range from 0.1 to 0.7 with a mean value close to 0.4. A similar trend is observed in the test set (Supplementary Fig. S3).

Tanimoto similarity analysis of molecular fingerprints for inhibitors showed that there is a wide chemical diversity on the test dataset for each CYP450 isoform. When noninhibitors of the test dataset are considered, Tanimoto coefficient values range from 0.1 to 0.8 (with a mean value close to 0.4), pointing out that, although the chemical diversity of the test compounds is still high, there are some groups of noninhibitors that belong in the same chemical space.

### RFE dictates which descriptors offer best CYP450 inhibitor/noninhibitor classification per CYP450 isoform

**FIG. 2.**
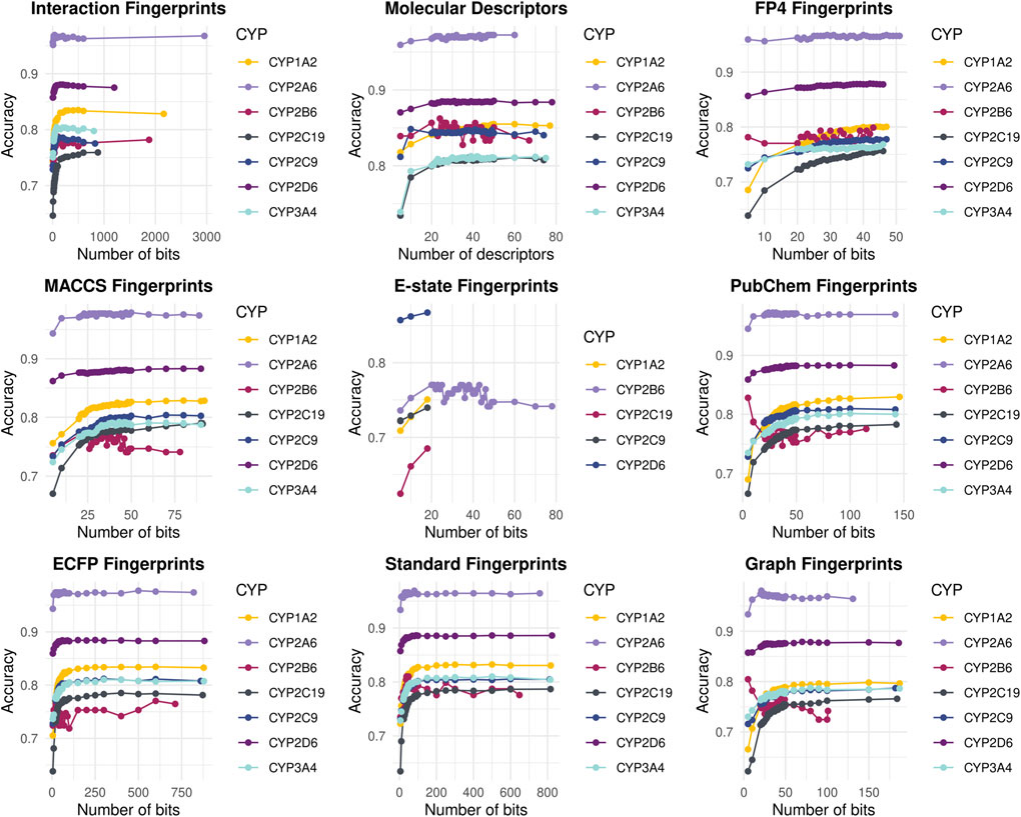
Search space of RFE selection process per feature type. Interaction Fingerprints. ECFP Fingerprints, Extended-Connectivity Fingerprints; E-state Fingerprints, Electrotopological state Fingerprints; MACCS Fingerprints, Molecular ACCess System Fingerprints; PLEC, (Protein-Ligand Extended Connectivity) Fingerprints.

To define which feature type provides the best discrimination ability, algorithms were trained with each feature type alone. The series of machine learning models developed for the binary classification of test compounds to inhibitors and noninhibitors exploited features of the generated interaction fingerprints and molecular descriptors, as well as different types of molecular fingerprints. After initial processing, removal of low variance and high correlation features, training sets were subjected to RFE per CYP450 isoform. RFE led to which bits (or descriptors) perform best per CYP450 isoform (Fig. 2).

CYP2A6 data enabled the highest discrimination ability in almost all feature types with different number of bits or descriptors per case. Yet, in the case of E-state fingerprints, no search was done as all the bits were filtered out for this feature type; the same for CYP2B6 and CYP3A4. Increasing the number of bits or descriptors did not result in better discrimination in most feature types and for all CYP450s. On the contrary, increasing feature dimensionality yielded lower accuracy. We suggest that feature selection is a vital step in a pipeline that aims to achieve high discrimination among inhibitors and noninhibitors. A total of *n* = 23 datasets per CYP450 isoform were produced, as summarized in Supplementary Table S5.

### A robust machine learning framework is introduced

**FIG. 3.**
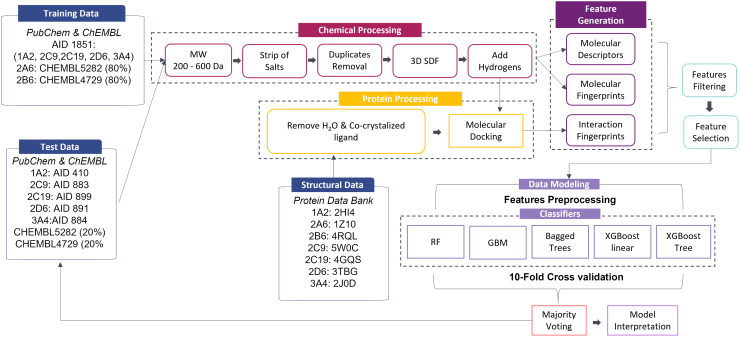
Workflow overview of the proposed framework.

An overview of the workflow developed and described herein is illustrated in Figure 3. The 10-fold cross-validation accuracy yielded by majority voting for each CYP450 isoform per feature type is depicted in Supplementary Figure S4. It is evident that when molecular descriptors were combined with molecular fingerprints, higher accuracy was achieved. Of note, the addition of interaction fingerprints on top of molecular descriptors and molecular fingerprints increased accuracy further (1–3% depending on the CYP450 isoform or 2–7%, when compared to molecular fingerprints alone). PLEC fingerprints exhibited an equal or even greater effect on prediction accuracy. Molecular descriptors performed equally or better than PLEC fingerprints upon 10-fold cross-validation.

As expected, the prediction accuracy of majority voting per feature type for each CYP450 isoform, when the external test set was considered, was lower due to data heterogeneity observed between training and external test sets (Supplementary Fig. S5). The increase in model accuracy that was obtained in the training data after the synergy of molecular fingerprints with molecular descriptors and interaction fingerprints still holds true only for CYP2B6 and CYP2C19. In the case of CYP1A2, the best performance is achieved by interaction fingerprints alone. For CYP3A4, model accuracy is higher when molecular descriptors are combined with molecular fingerprints alone. Finally, the models for CYP2A6 and CYP2D6 show the highest predictive accuracy based on molecular fingerprints alone. We suggest that such findings are crucial and shall be taken into account along with 10-fold cross-validation outcomes for the identification of the best performing feature types.

Comparing the performance per CYP450 isoform between the training and external test sets, optimal models can be derived. We define as optimal performance the case in which accuracy in both training and external test sets is high, yet devoid of a large difference (>20% may be a sign of overfitting). Similarly, feature types that yielded a better performance in the external test set than in the training set were also considered nonoptimal as they may be indicative of model underfitting. Overall, the optimal feature types per CYP450 isoform-related model are provided in Table 1.

**Table 1.** Optimal Performance Per Isoform for the Training and Test Sets

CYP450 isoform	Feature type	10-fold cross-validation					External test set				
		ACC (%)	SE (%)	SP (%)	MCC	AUC	ACC (%)	SE (%)	SP (%)	MCC	AUC
CYP1A2	PLEC	95.8	98.4	91.5	0.9	0.9	92.1	91.6	83.3	0.8	0.9
CYP2A6	ECFP	99.0	100	90.5	0.9	1	97.4	100	71.4	0.8	0.9
CYP2B6	PLEC+Desc.+Graph	90.2	100	66	0.8	0.99	88.1	87.5	63.6	0.7	0.9
CYP2C19	Desc.+Graph	93.9	94.3	91.9	0.9	0.99	76.7	57.1	60.9	0.4	0.8
CYP2C9	PLEC+Desc.+ECFP	96.4	99.9	87.9	0.9	0.9	81.3	38.4	33.7	0.25	0.73
CYP2D6	PLEC+Desc.+MACCS	92.2	100	45.5	0.7	0.9	89.2	64.5	43.5	0.47	0.88
CYP3A4	Desc.+PubChem	88.3	97.2	59.5	0.7	0.9	79.0	83.5	34.8	0.5	0.9

ACC, accuracy; SE, sensitivity; SP, specificity; MCC, Matthews correlation coefficient; AUC, area under curve; ECFP, Extended-Connectivity Fingerprints; PLEC, Protein-Ligand Extended Connectivity Fingerprints; Desc., descriptors; MACCS, Molecular ACCess System Fingerprints; FP4, FP4 Fingerprints; E-state, Electrotopological state Fingerprints; Desc., molecular descriptors; PubChem, PubChem fingerprints; Standard, Standard fingerprints; Graph, Graph fingerprints.

The performance of each feature type per CYP450 and evaluation metrics are depicted in the Supplementary Figures S6–S13 (for 10-fold cross-validation and external test sets). For each CYP450 isoform, the feature type, which achieved optimal performance, was determined based on accuracy, sensitivity, specificity, Matthews correlation coefficient, and AUC for both training and external test data, highlighting the generalization ability in each case.

For CYP1A2, the overall performance of interaction fingerprints alone indicated the robustness of the predictions and the generalization ability that this feature type provided for this isoform. Similar performances were obtained for CYP2A6 and CYP2B6 models. For CYP2A6, such performance was achieved with ECFP fingerprints alone. The CYP2B6 models were the net result of interaction fingerprints, molecular descriptors, and Graph fingerprints to achieve optimal performance and sufficient generalization. The CYP2C19 models had optimum performance employing only molecular descriptors and Graph fingerprints. An equivalent performance was observed for CYP2C9, CYP2D6, and CYP3A4 models. Models with best performance for CYP2C9 and CYP2D6 used interaction fingerprints combined with molecular descriptors and ECFP or MACCS fingerprints, while for CYP3A4, molecular descriptors and PubChem fingerprints were enough.

### CYP450 models share distinct significant variables from all feature types

**FIG. 4.**
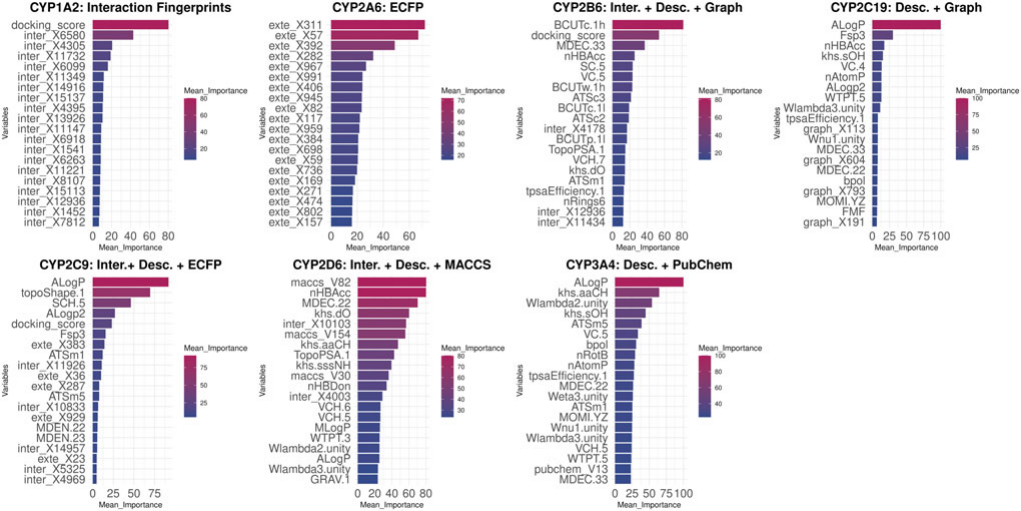
Top 20 features after ranking by their mean importance (majority voting scheme) per CYP450 isoform. Set of variables, ranked by importance for the discrimination of inhibitors versus noninhibitors for each CYP450 isoform. The twenty most important variables are depicted in each case. Interaction Fingerprints, Desc., Descriptors; PLEC fingerprints, (Protein-Ligand Extended Connectivity) Fingerprints; ECFP Fingerprints, Extended-Connectivity Fingerprints.

The interpretation of each machine learning model is an important step, as it explains the effect of the variables in the performance of the model and provides insights into the factors that are predicted to affect the inhibition of each CYP450 isoform. For each CYP450 isoform, the set of variables that was found to be optimal for the discrimination of the two classes is shown in Figure 4, where the top 20 most important variables are depicted.

For CYP1A2, besides the different bits, which were found to be significant, the most significant variable was the docking score with a contribution on the predictions equal to 80%. In the case of CYP2A6, we determined which bits from the ECFPs mostly affected model prediction. The models for CYP2B6 utilized interaction fingerprints, molecular descriptors, and graph fingerprints to produce optimal predictions, notwithstanding, most of the top 20 important features were molecular descriptors, with the docking score being the second most important variable. Interaction fingerprint bits were considered to be less important as they were ranked last.

For CYP2C19, optimal predictions were obtained when using molecular descriptors and graph fingerprints, but not interaction fingerprints. The models were affected by the ALogp parameter by almost 100%, which is justified by the fact that CYP2C19 inhibitors share structural features that include aromatic moieties, heterocycles, carbonyl groups, and aromatic nitrogen atoms (Beck et al., 2021). For CYP2C9 models, in which interaction fingerprints, molecular descriptors, and ECFPs were combined, we noticed that the descriptors ALogP and topoShape.1 were the two most important variables. Yet, the docking score was ranked fifth.

Similarly, CYP2D6 optimal models employed interaction fingerprints, molecular descriptors, and MACCS fingerprints. Physicochemical descriptors were among the most important features. The docking score was not an important feature for the CYP2D6 models and interaction bits had lower importance for this isoform. For CYP3A4, models employed a combination of molecular descriptors and PubChem fingerprints to achieve optimal performance. Once again, molecular descriptors had higher importance scores than the fingerprint bits. We point out that CYP450 models share distinct significant variables from all feature types.

## Discussion

There is a need to improve predictive models on CYP450 inhibition, which would bode well particularly for rational drug discovery and development, not to mention precision in drug repurposing.

Molecular fingerprints are widely used in the QSAR models as they consist of the descriptors of a molecule encoding its structural elements into a binary bit vector. They enable the understanding and prediction of the important chemical groups that may be responsible for inhibition or noninhibition. Molecular descriptors are numerical representations of the physicochemical properties of a molecule, used to characterize and compare its structure and behavior.

Interaction fingerprints, instead of describing the structure of a molecule, refer to the interactions between a molecule and a target protein. This type of representation focuses on the protein-ligand interactions at a molecular level, thus providing an insight on the structural features that are important for a molecule to have a biological effect on a specific target (Wójcikowski et al., 2019). By implementing such information from interaction fingerprints, we have explored the possibility of gaining better predictions for CYP450 inhibitors or noninhibitors (test compounds/ligands), applying enzyme structural information.

Herein, we took into account interaction fingerprints produced by docking calculations (training and external test sets) with the aim to add an extra level of information in machine learning models, including the morphology of CYP450's cavity as well as the predicted interactions between the test compounds (ligands) and the corresponding amino acid residues at the molecular level. Our final predictions are based on the morphological differences of each CYP450 cavity, together with the structural and physicochemical differences of the test compounds (ligands) (Bender et al., 2021). In machine learning, the addition of extra information does matter only when this information is useful. Intuitively, one would expect that adding the interaction information would achieve better performance for the models in question. According to our findings, this is not always the case.

Several computational approaches have been reported, where different methods have been implemented. To name, but a few as follows: the autoencoder neural network developed by Li et al. (2018); an XGBoost model, among various machine and deep learning classifiers by Wu et al. (2019); a GCNN model, which utilizes graph convolutional network with attention mechanism for feature extraction by Qiu et al. (2022); and a multitask learning approach by the iCYP-MFE framework (Nguyen-Vo et al., 2022).

Despite the overall benefits that multitask approaches provide, their main limitation is that a single input with a fixed type of features will not achieve optimal performance as our comparative analysis suggests. This is because each CYP450 isoform requires a different type of representation to achieve favorable discrimination, which is taken into account in our framework. Furthermore, deep learning approaches fail to give an insight into the important features employed for classification, even though they perform slightly better than machine learning models. Thus, our approach offers explainable models, although it may not provide optimum predictions. Our framework also incorporates a feature selection step, which removes data noise, reduces the number of features, and allows for model training time, while an extra safety layer is introduced to avoid overfitting. This step is missing from some of the aforementioned pipelines.

Our analysis, after taking into account the structural information of molecules (test compounds/ligands) and CYP450 isoforms, suggests that modeling smaller catalytic sites provides a better generalization ability of the corresponding models. This is evident in the models for CYP1A2, CYP2A6, and CYP2B6. These isoforms share small and narrow cavities, in which molecules are docked in specific orientations, and hence, the overall interactions of the test compounds (ligands) with the amino acid residues of the corresponding cavity are limited, leading to distinct patterns that are easy to be learned by the models during the training and cross-validation phase, achieving high accuracy results. CYP2C9, CYP2C19, CYP2D6, and CYP3A4 share larger cavity surface areas, allowing for more diverse interactions to occur. Thus, more complex patterns of interactions arise, challenging model generation and performance. Indeed, models had lower performance in the external test set for these isoforms.

There is no specific feature type that could generalize in all CYP450s due to their evolutionary structural variability. Such structural variability is reflected herein, not only when catalytic cavities are explored but also when CYP450 models share distinct significant variables from all feature types. We suggest that a robust framework should take into account different feature types per CYP450 isoform to achieve the best possible discrimination when inhibition is considered. Herein, robustness is achieved and may be also extended to efficacy predictions in drug repurposing schemes. Sitagliptin served as a paradigm. All features and functionalities have been included in our nonstop shop next-generation drug repurposing platform CloudScreen^®^.

## Conclusions

To our knowledge, the inhibitory/noninhibitory effects of molecules (test compounds/ligands) on CYP2A6 and CYP2B6 have not been extensively modeled. Herein, interaction fingerprints are taken into account to predict CYP450 inhibitors and noninhibitors for all seven major CYP450 isoforms. Our robust framework offers insights into how computational methods could better predict CYP450 inhibitors and how different types of molecular representation (molecular descriptors, molecular fingerprints, or protein-ligand interaction fingerprints) of test compounds can be fine-tuned to yield better results with higher accuracy.

## Supplementary Material

Supplemental data

Supplemental data

Supplemental data

Supplemental data

Supplemental data

Supplemental data

Supplemental data

Supplemental data

Supplemental data

Supplemental data

Supplemental data

## Data Availability

All data generated or analyzed during this study are included in this published article and its supplementary information files.

## Abbreviations Used

AUC: area under curve
CYP450: Cytochrome P450
ECFP: Fingerprints, Extended-Connectivity Fingerprints
E-state: Electrotopological state
GCNN: Geometric Convolutional Neural Network
MACCS Fingerprints: Molecular ACCess System Fingerprints
MCC: Matthews correlation coefficient
PLEC: Protein-Ligand Extended Connectivity
QSAR: quantitative structure-activity relationship
RFE: Recursive Feature Elimination
XGBoost: extreme gradient boosting
