# Supplementary material for: A Robust Machine Learning Framework Built Upon Molecular Representations Predicts CYP450 Inhibition: Toward Precision in Drug Repurposing
Source: OMICS. 2023 Jul 19;27(7):305–14. doi: 10.1089/omi.2023.0075 (PMC10357106; doi:10.1089/omi.2023.0075)
Supplement: Supplemental data [file Suppl_TableS1.docx]

**Table S1**. Dataset information for the test-compounds (ligands) explored

| **CYP450 isoform** | **Dataset** | **PubChem Bioassay Record ID** | **Number of inhibitors** | **Number of**  **non-inhibitors** |
| --- | --- | --- | --- | --- |
| CYP1A2 | Training | 1851 | 4355 | 6013 |
|  | Test | 410 | 156 | 326 |
| CYP2A6 | Training | Suppl. Table 2 | 63 | 555 |
|  | Test | Suppl. Table 2 | 14 | 138 |
| CYP2B6 | Training | Suppl. Table 3 | 50 | 124 |
|  | Test | Suppl. Table 3 | 11 | 31 |
| CYP2C9 | Training | 1851 | 2901 | 7292 |
|  | Test | 883 | 98 | 532 |
| CYP2C19 | Training | 1851 | 4949 | 6153 |
|  | Test | 884 | 184 | 487 |
| CYP2D6 | Training | 1851 | 1620 | 9740 |
|  | Test | 899 | 93 | 596 |
| CYP3A4 | Training | 1851 | 1588 | 4168 |
|  | Test | 891 | 584 | 1421 |

Sitagliptin does not inhibit nor induce CYP1A2, CYP2A6, CYP2B6, CYP2C9, CYP2C19, CYP2D6 and CYP3A4 human CYP450 isoenzymes and hence, served as a paradigm.
