# Supplementary material for: A Robust Machine Learning Framework Built Upon Molecular Representations Predicts CYP450 Inhibition: Toward Precision in Drug Repurposing
Source: OMICS. 2023 Jul 19;27(7):305–14. doi: 10.1089/omi.2023.0075 (PMC10357106; doi:10.1089/omi.2023.0075)
Supplement: Supplemental data [file Suppl_TableS2.docx]

**Table S2**. ChEMBL IDs for CYP2A6 assays.

| **Assay ID** | **Bio Assay Ontology** | **Assay ID** | **Bio Assay Ontology** | **Assay ID** | **Bio Assay Ontology** |
| --- | --- | --- | --- | --- | --- |
| CHEMBL839581 | single protein format | CHEMBL909371 | single protein format | CHEMBL1925455 | assay format |
| CHEMBL3390304 | single protein format | CHEMBL3706122 | assay format | CHEMBL4616442 | single protein format |
| CHEMBL1909133 | cell-based format | CHEMBL3528837 | assay format | CHEMBL2214080 | assay format |
| CHEMBL910617 | single protein format | CHEMBL4010498 | assay format | CHEMBL4370241 | single protein format |
| CHEMBL3745190 | assay format | CHEMBL2423312 | single protein format | CHEMBL1118622 | tissue-based format |
| CHEMBL1743368 | single protein format | CHEMBL1924633 | microsome format | CHEMBL3242644 | single protein format |
| CHEMBL3390307 | single protein format | CHEMBL900218 | single protein format | CHEMBL3889068 | single protein format |
| CHEMBL3390305 | single protein format | CHEMBL4422404 | microsome format | CHEMBL4122658 | single protein format |
| CHEMBL3390302 | single protein format | CHEMBL4016807 | assay format | CHEMBL4427251 | microsome format |
| CHEMBL4422431 | cell-based format | CHEMBL2416907 | microsome format | CHEMBL4033108 | assay format |
| CHEMBL4422408 | microsome format | CHEMBL4016359 | single protein format | CHEMBL4009082 | assay format |
| CHEMBL1743539 | single protein format | CHEMBL893809 | microsome format | CHEMBL1112474 | single protein format |
| CHEMBL1743367 | microsome format | CHEMBL4181971 | cell-based format | CHEMBL3539939 | assay format |
| CHEMBL3532211 | single protein format | CHEMBL896135 | single protein format | CHEMBL2149444 | single protein format |
| CHEMBL3423893 | single protein format | CHEMBL986384 | single protein format | CHEMBL4268627 | single protein format |
| CHEMBL3532209 | single protein format | CHEMBL4046787 | assay format | CHEMBL4627226 | single protein format |
| CHEMBL1924636 | microsome format | CHEMBL4181828 | single protein format |  |  |
| CHEMBL1743369 | single protein format | CHEMBL2354043 | single protein format |  |  |
| CHEMBL4150900 | microsome format | CHEMBL1924632 | microsome format |  |  |
| CHEMBL932656 | single protein format | CHEMBL3395910 | single protein format |  |  |
| CHEMBL832816 | single protein format | CHEMBL4144191 | microsome format |  |  |
| CHEMBL1023677 | single protein format | CHEMBL3100519 | assay format |  |  |
| CHEMBL2406772 | single protein format | CHEMBL899970 | single protein format |  |  |
| CHEMBL3705713 | single protein format | CHEMBL1821056 | single protein format |  |  |
| CHEMBL4010623 | assay format | CHEMBL4033440 | assay format |  |  |
| CHEMBL829411 | single protein format | CHEMBL2209445 | single protein format |  |  |
| CHEMBL4422401 | assay format | CHEMBL2183281 | single protein format |  |  |
| CHEMBL1260081 | single protein format | CHEMBL3776836 | tissue-based format |  |  |
