# Supplementary material for: A Robust Machine Learning Framework Built Upon Molecular Representations Predicts CYP450 Inhibition: Toward Precision in Drug Repurposing
Source: OMICS. 2023 Jul 19;27(7):305–14. doi: 10.1089/omi.2023.0075 (PMC10357106; doi:10.1089/omi.2023.0075)
Supplement: Supplemental data [file Suppl_TableS3.docx]

**Table S3**. ChEMBL IDs for CYP2B6 assays

| **Assay ID** | **Bio Assay Ontology** | **Assay ID** | **Bio Assay Ontology** | **Assay ID** | **Bio Assay Ontology** |
| --- | --- | --- | --- | --- | --- |
| CHEMBL910621 | single protein format | CHEMBL4406779 | microsome format | CHEMBL4616431 | single protein format |
| CHEMBL1743374 | single protein format | CHEMBL4039275 | assay format | CHEMBL3855757 | tissue-based format |
| CHEMBL1743376 | single protein format | CHEMBL4367267 | single protein format | CHEMBL3888370 | tissue-based format |
| CHEMBL1743384 | microsome format | CHEMBL2405000 | single protein format | CHEMBL3887127 | single protein format |
| CHEMBL1634992 | microsome format | CHEMBL4004717 | single protein format | CHEMBL3831049 | single protein format |
| CHEMBL1743372 | single protein format | CHEMBL1293110 | single protein format | CHEMBL3528838 | assay format |
| CHEMBL1743370 | single protein format | CHEMBL4016808 | assay format | CHEMBL3292471 | single protein format |
| CHEMBL1743371 | single protein format | CHEMBL4014089 | assay format | CHEMBL3825880 | single protein format |
| CHEMBL1743379 | single protein format | CHEMBL1032454 | single protein format | CHEMBL4144194 | microsome format |
| CHEMBL1743381 | single protein format | CHEMBL3242643 | single protein format | CHEMBL4406413 | microsome format |
| CHEMBL3531984 | assay format | CHEMBL1112475 | single protein format | CHEMBL4024916 | single protein format |
| CHEMBL1743378 | single protein format | CHEMBL4429413 | microsome format | CHEMBL3528848 | assay format |
| CHEMBL3534695 | assay format | CHEMBL4051509 | single protein format | CHEMBL3268503 | single protein format |
| CHEMBL3528881 | assay format | CHEMBL4009083 | assay format | CHEMBL4413480 | single protein format |
| CHEMBL4150902 | microsome format | CHEMBL4199851 | microsome format | CHEMBL1777405 | single protein format |
| CHEMBL989844 | microsome format | CHEMBL1292299 | single protein format | CHEMBL3624488 | single protein format |
| CHEMBL4304999 | microsome format | CHEMBL1292273 | single protein format | CHEMBL4197069 | microsome format |
| CHEMBL829412 | single protein format | CHEMBL3754961 | single protein format | CHEMBL2209443 | single protein format |
| CHEMBL1002234 | microsome format | CHEMBL3610819 | single protein format | CHEMBL3395911 | single protein format |
| CHEMBL3624808 | single protein format | CHEMBL4028667 | assay format | CHEMBL4236265 | single protein format |
| CHEMBL3636694 | assay format | CHEMBL4406416 | microsome format | CHEMBL2416906 | microsome format |
| CHEMBL4010568 | assay format | CHEMBL4416915 | single protein format | CHEMBL1260082 | single protein format |
| CHEMBL4010618 | assay format | CHEMBL4626212 | microsome format | CHEMBL3602022 | single protein format |
| CHEMBL999392 | single protein format | CHEMBL4007976 | assay format | CHEMBL4027672 | assay format |
| CHEMBL2173506 | single protein format | CHEMBL4263018 | single protein format | CHEMBL4433809 | single protein format |
| CHEMBL3766305 | tissue-based format | CHEMBL2173532 | single protein format | CHEMBL4414957 | microsome format |
| CHEMBL893810 | microsome format | CHEMBL3388334 | single protein format | CHEMBL4122659 | single protein format |
| CHEMBL4422411 | single protein format | CHEMBL3532642 | single protein format | CHEMBL3795703 | single protein format |
| CHEMBL2160814 | microsome format | CHEMBL1637352 | single protein format | CHEMBL4181829 | single protein format |
| CHEMBL1646223 | single protein format | CHEMBL4051181 | single protein format | CHEMBL4610964 | single protein format |
| CHEMBL869680 | single protein format | CHEMBL4267017 | single protein format | CHEMBL4370242 | single protein format |
| CHEMBL2020718 | microsome format | CHEMBL4001262 | single protein format | CHEMBL4132510 | single protein format |
| CHEMBL3707545 | assay format | CHEMBL4158405 | microsome format | CHEMBL3744267 | single protein format |
| CHEMBL2439728 | single protein format | CHEMBL4265241 | single protein format | CHEMBL4149891 | microsome format |
| CHEMBL3887496 | single protein format | CHEMBL4404457 | microsome format | CHEMBL3528879 | assay format |
| CHEMBL3100518 | assay format | CHEMBL4016548 | single protein format | CHEMBL4200935 | microsome format |
| CHEMBL1664603 | single protein format | CHEMBL4031527 | assay format | CHEMBL4040890 | single protein format |
| CHEMBL2039546 | single protein format | CHEMBL4016360 | single protein format | CHEMBL3803542 | single protein format |
| CHEMBL930255 | single protein format | CHEMBL4351544 | single protein format | CHEMBL3539940 | assay format |
| CHEMBL2354042 | single protein format | CHEMBL1925456 | assay format | CHEMBL4033441 | assay format |
| CHEMBL3608070 | single protein format | CHEMBL4181972 | cell-based format | CHEMBL4412517 | microsome format |
| CHEMBL3888163 | tissue-based format | CHEMBL3614364 | single protein format | CHEMBL3637617 | single protein format |

**Table S3**. ChEMBL IDs for CYP2B6 assays (cont.)

| **Assay ID** | **Bio Assay Ontology** | **Assay ID** | **Bio Assay Ontology** | **Assay ID** | **Bio Assay Ontology** |
| --- | --- | --- | --- | --- | --- |
| CHEMBL4345452 | microsome format | CHEMBL1063238 | single protein format | CHEMBL3858945 | single protein format |
| CHEMBL2183280 | single protein format | CHEMBL4390470 | single protein format | CHEMBL4426271 | single protein format |
| CHEMBL4404737 | single protein format | CHEMBL1262357 | microsome format | CHEMBL3607801 | assay format |
| CHEMBL4373232 | microsome format | CHEMBL4424325 | microsome format | CHEMBL4619288 | single protein format |
| CHEMBL4382121 | microsome format | CHEMBL4388568 | single protein format | CHEMBL4385837 | microsome format |
| CHEMBL4627227 | single protein format | CHEMBL4611433 | single protein format | CHEMBL4606258 | microsome format |
| CHEMBL3795104 | single protein format |  |  |  |  |
