# Supplementary material for: A Robust Machine Learning Framework Built Upon Molecular Representations Predicts CYP450 Inhibition: Toward Precision in Drug Repurposing
Source: OMICS. 2023 Jul 19;27(7):305–14. doi: 10.1089/omi.2023.0075 (PMC10357106; doi:10.1089/omi.2023.0075)
Supplement: Supplemental data [file Suppl_FigureS1.docx]

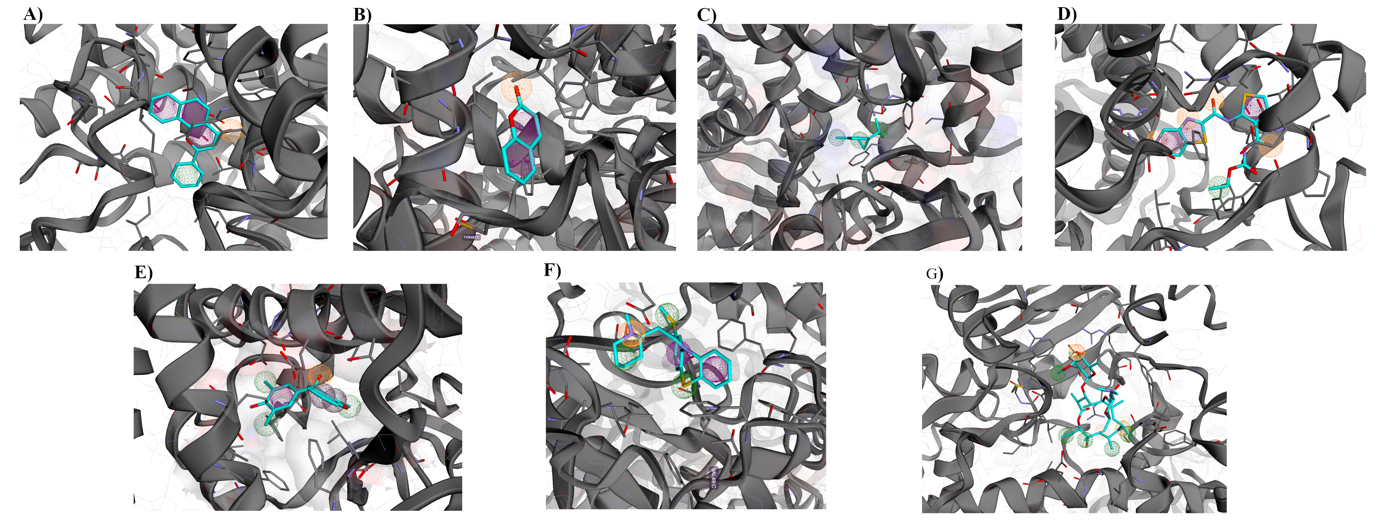


**Fig. S1**. Structure-based pharmacophore modeling analysis on the co-crystalized ligands for each of the CYP450 isoforms to explore further their catalytic cavity. A) CYP1A2, B) CYP2A6, C) CYP2B6, D) CYP2C9, E) CYP2C19, F) CYP2D6 and G) CYP3A4.
