# Supplementary material for: A Robust Machine Learning Framework Built Upon Molecular Representations Predicts CYP450 Inhibition: Toward Precision in Drug Repurposing
Source: OMICS. 2023 Jul 19;27(7):305–14. doi: 10.1089/omi.2023.0075 (PMC10357106; doi:10.1089/omi.2023.0075)
Supplement: Supplemental data [file Suppl_TableS4.docx]

**Table S4**. Pharmacophore features per CYP450 isoform

| **CYP450 isoforms** | **Pharmacophore Features** | | | |
| --- | --- | --- | --- | --- |
|  | **Aromatic** | **Hydrophobic** | **Hydrogen Acceptor** | **Hydrogen Donor** |
| CYP1A2 | 4 | 1 | 1 | 0 |
| CYP2A6 | 2 | 0 | 1 | 0 |
| CYP2B6 | 0 | 3 | 0 | 0 |
| CYP2C9 | 3 | 1 | 4 | 0 |
| CYP2C19 | 1 | 5 | 1 | 0 |
| CYP2D6 | 2 | 3 | 1 | 0 |
| CYP3A4 | 0 | 5 | 2 | 1 |
