# Supplementary material for: A Robust Machine Learning Framework Built Upon Molecular Representations Predicts CYP450 Inhibition: Toward Precision in Drug Repurposing
Source: OMICS. 2023 Jul 19;27(7):305–14. doi: 10.1089/omi.2023.0075 (PMC10357106; doi:10.1089/omi.2023.0075)
Supplement: Supplemental data [file Suppl_FigureS3.docx]

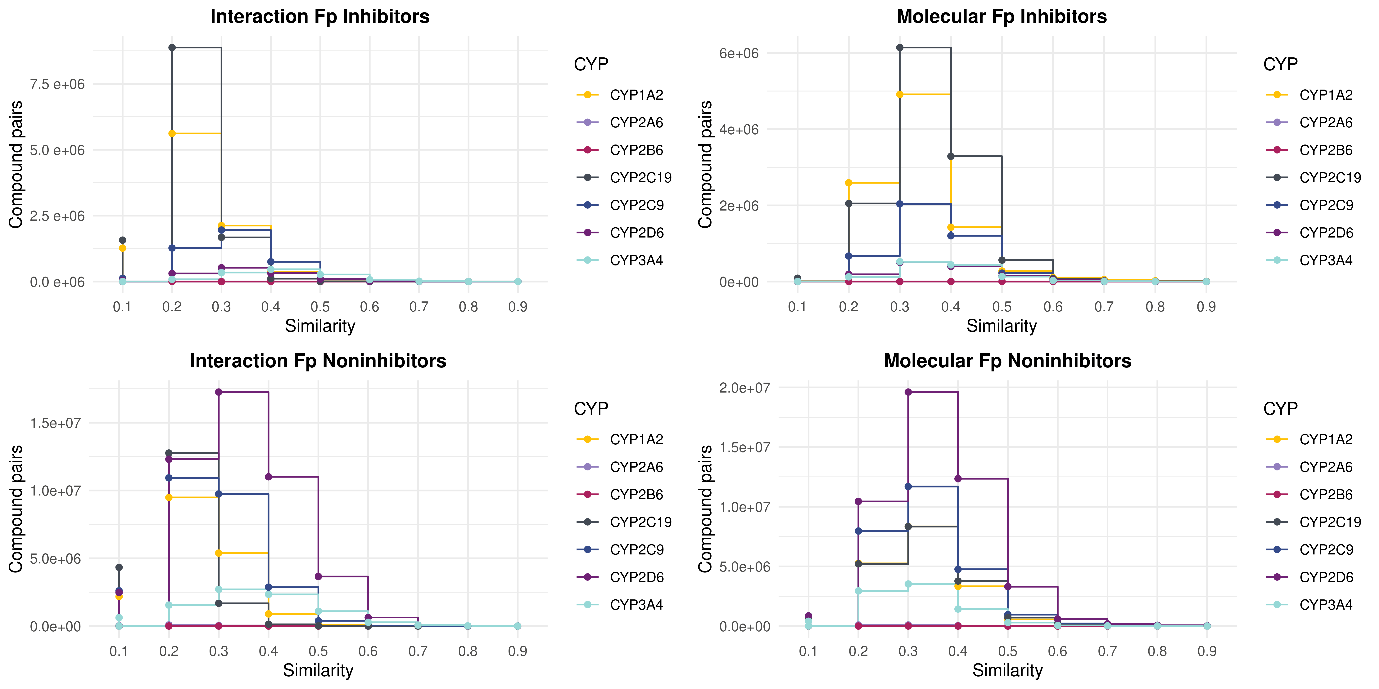


**Fig. S3**. Similarity profiles of the test set compounds, computed for molecular and interaction fingerprints. Interaction Fp, Interaction Fingerprints; Molecular Fp, Molecular Fingerprints
