# Supplementary material for: A Robust Machine Learning Framework Built Upon Molecular Representations Predicts CYP450 Inhibition: Toward Precision in Drug Repurposing
Source: OMICS. 2023 Jul 19;27(7):305–14. doi: 10.1089/omi.2023.0075 (PMC10357106; doi:10.1089/omi.2023.0075)
Supplement: Supplemental data [file Suppl_TableS5.docx]

**Table S5**. Molecular descriptors/fingerprints and interaction fingerprints per CYP450 isoform following filtering and feature selection.

| **Feature Type** | **CYP450 isoforms** | | | | | | |
| --- | --- | --- | --- | --- | --- | --- | --- |
|  | CYP1A2 | CYP2A6 | CYP2B6 | CYP2C9 | CYP2C19 | CYP2D6 | CYP3A4 |
| PLEC | 500 | 39 | 80 | 200 | 882 | 150 | 80 |
| Desc. | 50 | 48 | 24 | 10 | 38 | 50 | 39 |
| FP4 | 44 | 40 | 43 | 41 | 45 | 42 | 46 |
| MACCS | 80 | 47 | 20 | 70 | 90 | 80 | 43 |
| E-state | 18 | - | - | 18 | 18 | 18 | - |
| PubChem | 146 | 24 | 5 | 100 | 143 | 100 | 100 |
| ECFP | 600 | 500 | 600 | 300 | 400 | 250 | 300 |
| Standard | 500 | 80 | 38 | 600 | 816 | 90 | 500 |
| Graph | 150 | 21 | 5 | 182 | 184 | 70 | 80 |
| Desc. + FP4 | 94 | 88 | 67 | 51 | 83 | 92 | 85 |
| Desc. + MACCS | 130 | 95 | 44 | 80 | 128 | 130 | 82 |
| Desc. + E-state | 68 | - | - | 28 | 56 | 68 | - |
| Desc. + PubChem | 196 | 72 | 29 | 110 | 181 | 150 | 139 |
| Desc. + ECFP | 650 | 548 | 624 | 310 | 438 | 300 | 339 |
| Desc. + Standard | 550 | 128 | 62 | 610 | 854 | 140 | 539 |
| Desc. + Graph | 200 | 69 | 29 | 192 | 222 | 120 | 119 |
| PLEC + Desc. + FP4 | 594 | 127 | 147 | 251 | 965 | 242 | 165 |
| PLEC + Desc.  +MACCS | 630 | 134 | 124 | 280 | 1010 | 280 | 162 |
| PLEC+ Desc.  + E-state | 568 | - | - | 228 | 938 | 218 | - |
| PLEC + Desc.  + PubChem | 696 | 111 | 109 | 310 | 1063 | 300 | 219 |
| PLEC + Desc.  + ECFP | 1150 | 587 | 704 | 510 | 1320 | 450 | 419 |
| PLEC + Desc.  + Standard | 1050 | 167 | 142 | 810 | 1736 | 290 | 619 |
| PLEC + Desc.  + Graph | 700 | 108 | 109 | 392 | 1104 | 270 | 199 |

ECFP, Extended-Connectivity Fingerprints; PLEC, Protein-Ligand Extended Connectivity Fingerprints; Desc., descriptors; MACCS (Molecular ACCess System) Fingerprints; FP4, FP4 Fingerprints; E-state, Electrotopological state Fingerprints; Desc., molecular descriptors; PubChem, PubChem fingerprints; Standard, Standard fingerprints; Graph, Graph fingerprints.
