# Supplementary material for: A Robust Machine Learning Framework Built Upon Molecular Representations Predicts CYP450 Inhibition: Toward Precision in Drug Repurposing
Source: OMICS. 2023 Jul 19;27(7):305–14. doi: 10.1089/omi.2023.0075 (PMC10357106; doi:10.1089/omi.2023.0075)
Supplement: Supplemental data [file Suppl_FigureS5.docx]

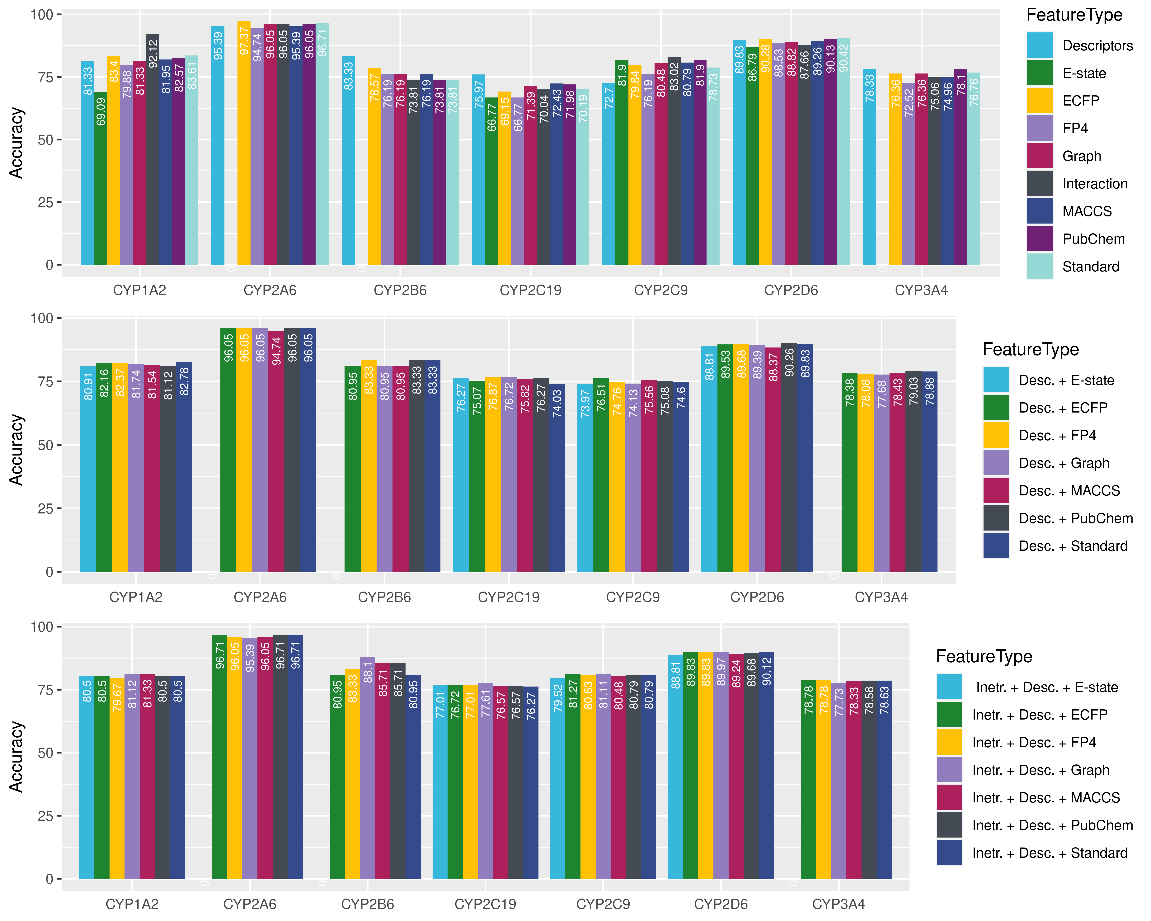


**Fig. S5**. Accuracy of the five classifiers majority voting per feature type and CYP450 isoform (external test-set)
